# Discrimination Between Cervical Cancer Cells and Normal Cervical Cells Based on Longitudinal Elasticity Using Atomic Force Microscopy

**DOI:** 10.1186/s11671-015-1174-y

**Published:** 2015-12-14

**Authors:** Xueqin Zhao, Yunxin Zhong, Ting Ye, Dajing Wang, Bingwei Mao

**Affiliations:** College of Life Sciences, Zhejiang Sci-Tech University, Hangzhou, 310018 People’s Republic of China; State Key Laboratory of Physical Chemistry of the Solid Surfaces, Department of Chemistry, College of Chemistry and Chemical Engineering, Xiamen University, Xiamen, 361005 People’s Republic of China

**Keywords:** Cell mechanics, Nanoindentation, Atomic force microscopy, Longitudinal elasticity, Cervical cancer

## Abstract

The mechanical properties of cells are considered promising biomarkers for the early diagnosis of cancer. Recently, atomic force microscopy (AFM)-based nanoindentation technology has been utilized for the examination of cell cortex mechanics in order to distinguish malignant cells from normal cells. However, few attempts to evaluate the biomechanical properties of cells have focused on the quantification of the non-homogeneous longitudinal elasticity of cellular structures. In the present study, we applied a variation of the method of Carl and Schillers to investigate the differences between longitudinal elasticity of human cervical squamous carcinoma cells (CaSki) and normal cervical epithelial cells (CRL2614) using AFM. The results reveal a three-layer heterogeneous structure in the probing volume of both cell types studied. CaSki cells exhibited a lower whole-cell stiffness and a softer nuclei zone compared to the normal counterpart cells. Moreover, a better differentiated cytoskeleton was found in the inner cytoplasm/nuclei zone of the normal CRL2614 cells, whereas a deeper cytoskeletal distribution was observed in the probing volume of the cancerous counterparts. The sensitive cortical panel of CaSki cells, with a modulus of 0.35~0.47 kPa, was located at 237~225 nm; in normal cells, the elasticity was 1.20~1.32 kPa at 113~128 nm. The present improved method may be validated using the conventional Hertz–Sneddon method, which is widely reported in the literature. In conclusion, our results enable the quantification of the heterogeneous longitudinal elasticity of cancer cells, in particular the correlation with the corresponding depth. Preliminary results indicate that our method may potentially be applied to improve the detection of cancerous cells and provide insights into the pathophysiology of the disease.

**Electronic supplementary material:**

The online version of this article (doi:10.1186/s11671-015-1174-y) contains supplementary material, which is available to authorized users.

## Background

Cancer is currently one of the leading causes of death worldwide. Tumorigenesis and oncogenic progression not only cause biological and functional alterations but also result in mechanical and structural abnormalities in cells. Pathophysiology studies suggest that the etiology of many human diseases is related to deviation from the normal structural and mechanical properties of cells, as well as to abnormal mechanotransduction [1, 2]. Several studies have reported that alterations in the mechanical properties of cells and the extracellular matrix are responsible for cancer progression [3, 4]. Currently, the gold standard for diagnosis of most solid tumors is based on tissue biological changes or specific antibody labeling of tissue specimens. However, the diagnosis of cancer based on morphological examination is not always accurate, as the morphology of malignant cells often resembles the common ones and it depends on the physician’ skill and knowledge. As such, research into the biomechanics of cancer cells is expected to contribute to the elucidation of disease pathophysiology and the discrimination between normal and malignant cells [5].

A variety of techniques, such as atomic force microscopy (AFM), optical magnetic twisting cytometry, laser-tracking microrheology, optical tweezers, and micropipette aspiration, have been used to probe the mechanical properties of normal and malignant cells [6–8]. In particular, AFM techniques with piconewton sensitivity and nanometer lateral resolution enable real-time biomechanical measurements in the action of a chemical, mechanical, or physiological process [9–12]. A comparison of the mechanical properties reveals that cancerous cells and tissue isolated from cancer patients are softer and more deformed [13–15]. In order to examine these mechanical properties, the relative Young’s modulus is typically determined for indentation depths within the range of 200–400 nm, which encompasses the cell cortex (the 50–100-nm-thick zone below the plasma membrane).

However, mammalian cells are highly discrete, heterogeneous structures that possess spatially varying elasticity and cell heights, as well as a heterogeneous underlying cytoskeleton. In addition, dynamic local remodeling of the cell surface architecture occurs on an ongoing basis during physiological processes [16]. Berdyyeva et al. noted that the Hertz–Sneddon model could not be applied beyond the penetration depth of 250 nm for the cell edge and ~100 nm for other parts of the cell [17]. Pogoda et al. performed a depth-sensing analysis of the mechanical characteristics of fibroblasts, in which only the cell cortex was probed for small indentation depths (about 200 nm) and the overall stiffness of the whole cell was obtained for large indentation depths (about 1400 nm) [18]. In addition, Ramos et al. demonstrated that the goodness of fit for Hertz models decreases with increasing indentation depth [19]. Although greater depths were probed in these studies, only one averaged elastic value was used to characterize the overall mechanics of the cells, which may have resulted in the underestimation of the heterogeneity of the cellular cytoskeleton. Moreover, data on the corresponding depths, which is expected to provide insights into cancer pathophysiology and enable early diagnosis of disease, have not been reported to date. Carl and Schillers sectioned single linearized force curves based on slopes and found two district elasticity zones; however, only one curve was analyzed and presented [20]. Obtaining and analyzing substantial data by the method of Schillers et al. is necessary to achieve a more reliable quantification of the mechanical properties of cells.

In the present study, human cervical cancer cells (CaSki cells) and normal cervical epithelial cells (CRL2614) were used to investigate the longitudinal elasticity in regions of the cell underlying the cell cortex. Substantial force curves were obtained and analyzed using data obtained by the method of Schillers et al. Then, Gauss fitting was applied to examine the elastic distribution and corresponding depth. We attempted to correlate disease state and subsequent changes in longitudinal elasticity. The present method was quantitatively validated by the conventional Hertz–Sneddon method, which is widely reported in the literature. In addition, the topography of both cell lines was investigated by AFM in order to identify and analyze qualitative changes associated with cancer (see Additional file 1). Our findings are expected to contribute to cancer diagnostics and elucidation of the pathophysiology of the disease.

## Methods

### Cell Culture

The normal human cervical cell line (CRL2614) and malignant human cervical carcinoma cells (CaSki) were used in this study. For the purpose of routine culture, both cell lines were incubated at 37 °C under 5 % CO_2_ and 95 % humidity. CaSki cells were cultured in Dulbecco’s modified Eagle’s medium (DMEM) with 10 % fetal bovine serum (*v*/*v*, Hyclone). CRL2614 cells were grown in keratinocyte serum-free medium (K-SFM). For AFM indentation and imaging, cells were seeded and cultured in a petri dish of 90-mm diameter. The cell density was controlled to ensure that elasticity measurements were performed at the single-cell level. Cells were washed three times with PBS before AFM measurements. Then, the cell medium was replaced by a serum-free medium and cells were incubated for 30 min at 37 °C under 5 % CO_2_ and 95 % humidity.

### AFM Measurements

**Fig. 1:**
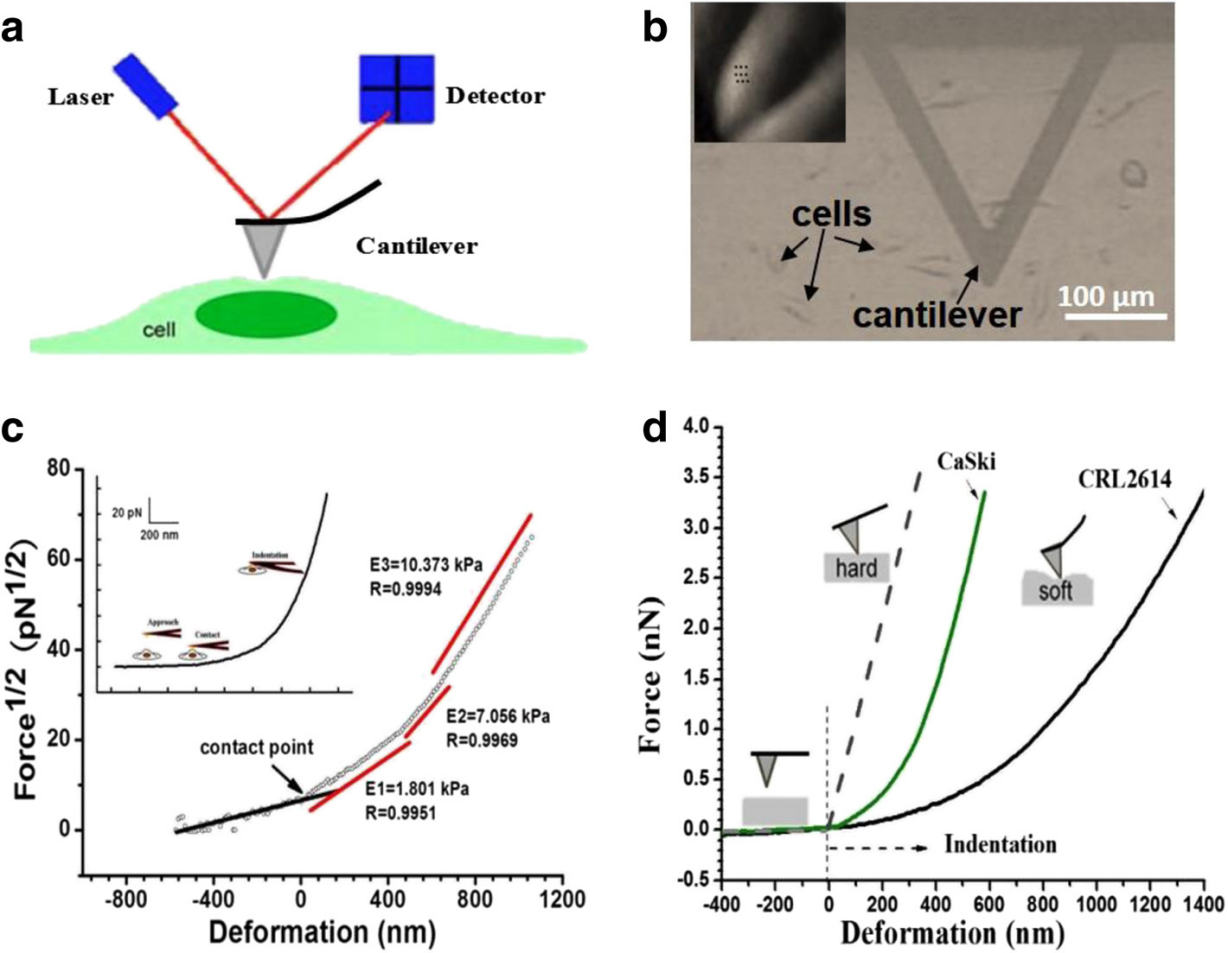
Schematic of the AFM elasticity measurements. **a** Illustration of cell elasticity measurements using AFM. **b** The optical image from the top-view camera integrated with the AFM system used. *Inset*: an illustration of the force spectroscopy mode; a grid has been set over the cell nucleus (CaSki cells, *deflection image*). **c** Force at the power 1/2 versus deformation relationship of a cell. Such representation (Eq. 1) of the data reveals three linear regimes (E1, E2, and E3) of the curve (*red lines* correspond to the fits; *black circles* represent the experimental data). The *inset* shows the force–deformation curve of the same indentation data. **d** Young’s modulus is determined by subtraction of curves recorded on hard (Si sheet) and soft surfaces (CaSki and CRL2614 cells)

Force measurements and AFM imaging were performed on an Agilent 5500ILM SPM (Agilent Technologies, Inc., USA) equipped with a N9524A scanner and video system (Fig. 1a).V-shaped silicon nitride cantilevers (Veeco, USA) with a nominal spring constant of 6 pN/nm were used in all the experiments. The spring constants of the cantilevers were calibrated by the thermal tune method [21].

Prior to AFM measurements, the sensitivity of the photodetector was measured by detecting the force response against a piece of silicon wafer placed directly in the sample plate [22]. Force curves captured over the nucleus region of a single intact cell, which is less affected by substrate stiffness. Only the approach curves were used to calculate elasticity. Each cell was probed by AFM at several locations over a 10 × 10 mm area (Fig. 1b). No less than 10 cells were measured and 15 force curves were recorded at different positions on each cell. For each dish, measurements were performed within a 1-h period as our own observations and other reports [23] indicate that cells remain viable for this duration. A constant approach velocity of 0.5 μm/s was chosen.

### Data Processing and Analysis

The force-indentation curve was obtained by subtracting the force curves recorded on a silicon wafer surface from those recorded on the cell surface. In order to obtain force-indentation curves, two measurement depths, namely shallow indentation and deep indentation, were selected. The shallow indentation depth reached the cellar cortex and was characterized as a constant value, whereas deep indentation, which probed 1500 nm under the cell membrane, was used for depth-sensing analysis of longitudinal elasticity above the cell nucleus. The cell height of fixed Hela cell was in the range of 3.5–4.9 μm measured by AFM [24].

For shallow indentation, the approach section of F-D curve was fit to the Hertz–Sneddon model with the cone [15]. The Young’s modulus was calculated for a constant indentation depth of 200 nm using semi-automatic processing software developed by Shi et al. [25]

1 $$ F={\delta}^2\frac{2}{\pi}\frac{E}{1-{v}^2} \tan \partial $$

where *F* is the loading force, *δ* is the indentation depth, *v* is the Poisson’s ratio, ∂ is the half-opening angle of the AFM tip, and *E* is the local Young’s elastic modulus to be determined. Poisson’s ratio was assumed to 0.5, as cells may be treated as incompressible material.

For deep indentation (indentation depths of up to 1500 nm), we processed F-D curves according to the method of Schillers et al. However, some improvements were made to achieve a more reliable quantification of the depth-sensing mechanical properties of the cells. First, V-shaped silicon nitride cantilevers displaced the colloidal probe to sensitively touch the cells. Next, substantial F-D data were obtained from not less than 10 cells, which ensured reproducibility of the results. Then, histograms and Gaussian fits were introduced to process the data. To summarize, firstly, Eq. 1 was transformed into the linearized dependence of the deformation on the force by taking the power 1/2 on both sides of the equation. Secondly, the indentation data were plotted according to the linearized form of transformed Eq. 1, ensuring that the linear regression of each portion was above 99.5 %. Then, various linear slopes and the corresponding depths were recorded and calculated. Finally, multi-peak Gaussian fits of the histograms of the elasticity and indentation depth were performed using origin 7.5 software. The most probable values were determined and expressed as means ± standard deviation (SD).

## Results and Discussion

### Single Curve Analysis

The elasticity modulus determined using a spherical probe represents the average elastic response of the cell, whereas a sharp tip is capable of touching the surface right between the cytoskeletal fibers, or directly on the top of the fibers, thereby substantially increasing the extent to which the heterogeneity of local elastic properties may be elucidated. Hence, the use of cone tips to probe cell elasticity of the cell enables clear discrimination between the properties of cells, at both superficial and high depths. Moreover, the apparent stiffness remains relatively constant below 415 nm/s but increases monotonically at higher approach velocities [26]. Low probe velocities minimize viscous losses. Measurements are dominated by elastic behavior at probe velocities below 1 μm/s; however, a very slow process may easily induce a non-trivial biological response [27]. Therefore, for the studied cells, a constant approach velocity of 0.5 μm/s was chosen.

A representative of the linearized form of the Hertz model is shown in Fig. 1c. Linear regression revealed three linear slopes, suggesting that the heterogeneous structures of the cell showed three layers, in terms of mechanical properties, in the probing volume. These multilayered structures were also observed by Kasas et al., Schiller and Fässler, and Pogoda et al., who reported that the first layer (the most superficial) represents the cortical actin cytoskeleton, the second layer represents the intermediate filament and microtubule network, and the third layer represents the nuclear zone [18, 28, 29]. Direct studies focusing on the cell nucleus found that this organelle shows higher stiffness than the cytoplasm [30, 31]. Our results indicate that as the probe approached the nucleus from the cell surface, the elastic moduli of the three domains were found to increase successively, and above 70 % of the measurements showed that the elastic moduli of the third layer (defined as E3) are larger than those of the second layer (defined as E2). Figure 1d represents a typical F-D curve for the normal CRL2614 cells and the cancerous CaSki cells. It is evident the relationship between force and indentation depth increases with higher force in CaSki cells, indicating a larger deformability of living cancerous cells.

### Shallow Indentation Analysis

The elastic properties of cells are best described by the elastic modulus. The obtained Young’s modulus values depend on various factors, such as the substrate used for cell growth, loading rate, indentation depth, or cultivation period [32]. Assuring the same conditions can provide better comparison between studied samples, which can give fundamental insights into disease progression.

**Fig. 2:**
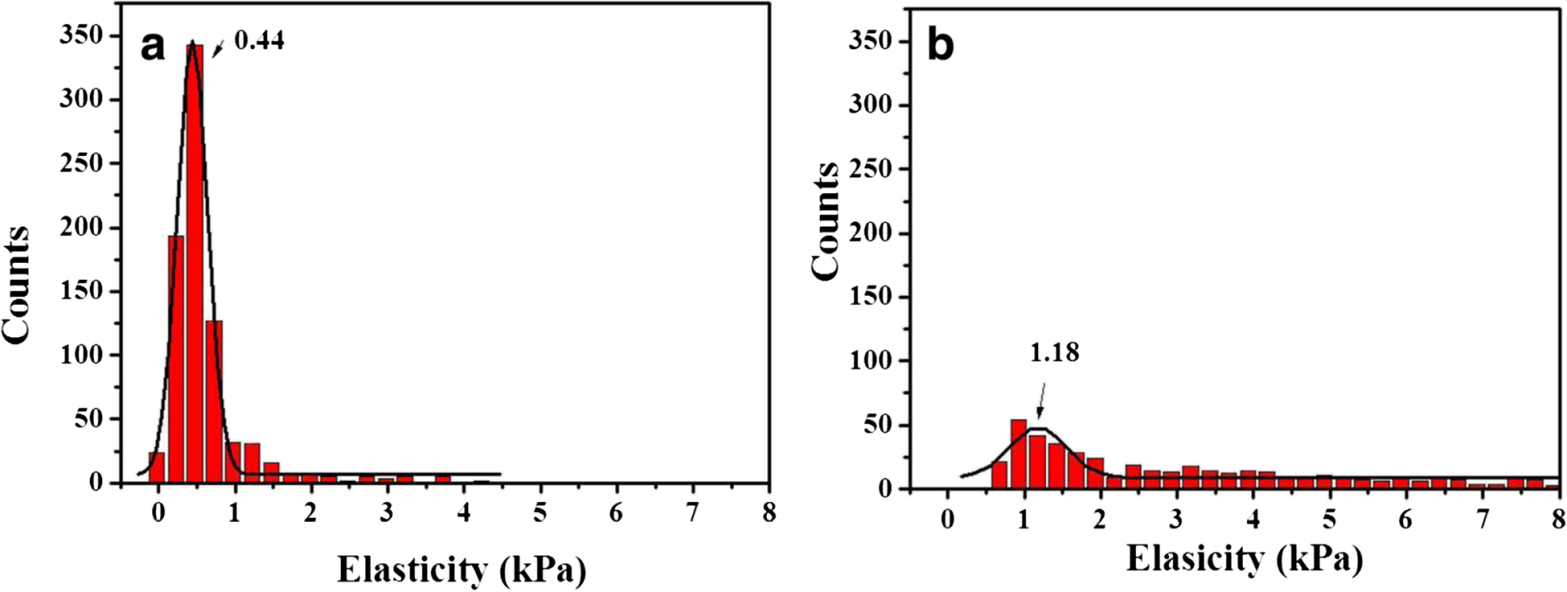
Distribution of Young’s moduli at shallow indentations (<200 nm) for **a** human cervical cancer cells (CaSki) and **b** normal cervical epithelial cells (CRL2614). Histogram bin width of 250 Pa. Gaussian fitting was performed for peak elasticity (*solid line*)

The cell cortex is the first significant mechanical compartment within the cell. The relative stiffness of the cortex is consistent with the observed increase in F-actin polymerization and, consequently, in F-actin concentration [33]. Shallow indentation measurements were performed to probe cortical stiffness. This method has been widely used for analyzing cell elasticity, and our data was comparable with results reported in the literature. As shown in Fig. 2, CaSki cells with a Young’s modulus of 0.44 ± 0.06 kPa exhibited a more deformable cortex relative to CRL2614 cells, indicating an irregular organization of actin filaments within the cortex of CaSki cells. Moreover, the distribution of the Young’s modulus for CRL2614 cells was nearly twofold broader than that for CaSki cells due to the higher variation of the cytoskeletal density. These results are in agreement with data reported in similar studies of breast cancer [17], cervical squamous carcinoma [34], oral squamous cell carcinoma [35], and ovarian cancer [27, 36].

### Deep Indentation Analysis

**Fig. 3:**
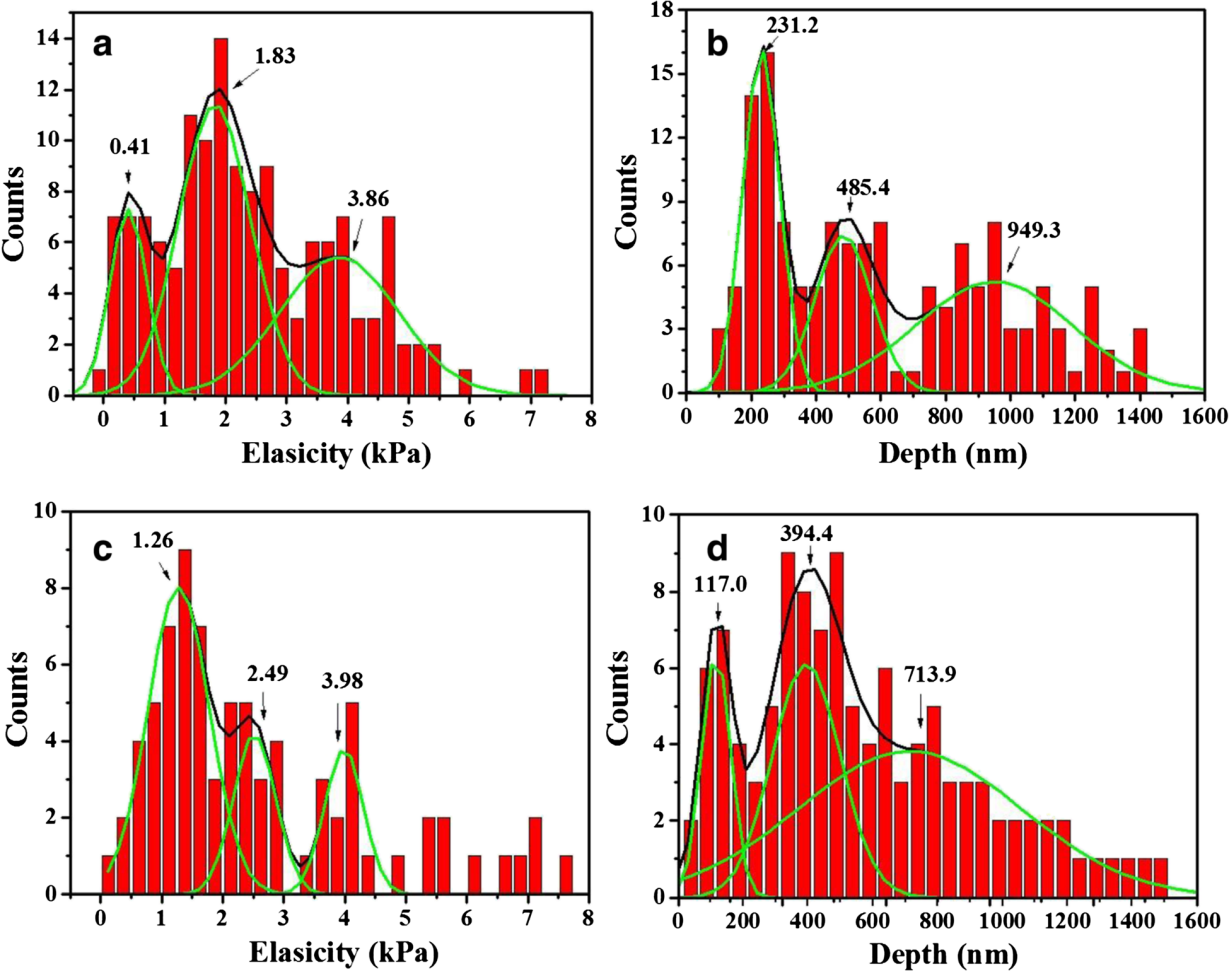
Histograms showing Gaussian fittings of Young’s moduli and corresponding depth for cells studied. **a**, **c** Young’s moduli and **b**, **d** corresponding depth, **a**, **c** for human cervical cancer cells (CaSki) and **b**, **d** for normal cervical epithelial cells (CRL2614). The *outlined data* refers to the most probable values. Histogram bin widths of 500 Pa and 50 nm, respectively

Deep indentation enabled the analysis of heterogeneity to a greater extent. A clear distinction between the surface and inner structures provides valuable indications about the components of the cytoskeleton, which may be affected by pathological processes. Hence, by performing deep indentation measurements (below 1500 nm), a change in the longitudinal elasticity due to pathophysiological alterations may be examined, which is potentially of significance for the diagnosis and elucidation of the pathogenesis of cancer. Histograms representing longitudinal elasticity and panel depth are presented in Fig. 3. As the representative linearized curve shows three distinct linear slopes (Fig. 1c), we decomposed the observed distribution into three Gaussian curves using three-peak fitting. Combining this data with the results of the analysis for the linearized form of the force curve (Fig. 1d), we speculated that the three legible peaks in Fig. 3a, b sequentially represent three panels of elasticity, from the cell surface to the interior of the cell.

In order to quantify the distribution effect of the longitudinal panels, we analyzed peak resolution *R* and relative amount A%. *R* was defined as the ratio between the distance of peak values and the sum of peak widths at half height, with the second peak as reference. A% was defined as the ratio between single peak area and total area, for elasticity At% and depth Ad%. All parameters from the two measurements (shallow indentation and deep indentation) are shown in Table 1. As shown in Fig. 3a and 3c, CRL2614 cells displayed better cytoskeleton differentiation in the inner panels of the probing volume (the last two panels) than the cancerous CaSki cells, for which *R* was increased by 54 %. Comparison of area percentages of elasticity panels revealed that in CRL2614 cells, the cortex accounted for over half of the cytoskeleton (At% 57 %), whereas in CaSki cells, the intermediate filaments and microtubule network constituted a significant proportion of the cytoskeleton (At% 47 %). Analysis of depth distribution (shown in Fig. 3b and 3d) revealed that CRL2614 cells had a low *R* of the inner panels (*R* 0.07) and high Ad% of the third panel (Ad% 59 %), which manifested as a highly overlapping cytoskeleton underlying the cortex. However, CaSki cells showed a twofold increase in *R* of the inner panels as compared with CRL2614 cells, indicating a relatively independent and concentrated distribution of the inner panels.

**Table 1:** The effects of normal cervical cells CRL2614 and cancer cells CaSki on cell elasticity and depth distribution

	Elasticity				Depth				Density	
	Mean	SD	Ae%	*R*	Mean	SD	Ad%	*R*	*D*	D%
CaSki	0.41	0.065	15.54	1.59	231.19	6.11	32.28	1.76	0.48	14.24
	1.83	0.08	47.4	0	485.39	17.53	22.98	0	2.06	61.13
	3.86	0.28	37.05	1.31	949.26	47.72	44.74	1.41	0.83	24.63
CRL2614	1.26	0.062	57.22	1.5	116.95	6.5	12.68	1.86	04.51	79.26
	2.49	0.091	24.9	0	394.38	11.36	28.73	0	0.87	15.29
	3.98	0.09	17.88	2.05	713.92	85.1	58.59	0.71	0.31	5.45

Rows of data came from Gaussian distribution done

The relative density of the cytoskeleton panel in the probing volume may be expressed as the ratio of At% to Ad%, represented as *D*. As shown in Table 1, the cancerous CaSki cells showed at least a threefold increase in elastic density (D%) of the inner panels; however, the elastic density of the cortical panel decreased by 82 % compared with its normal counterparts. These finding may be attributable to the depolymerization of F-actin at the cell cortex [33] and upregulation of the expression of microtubulin in the inner panels [37]. Changes in cell stiffness are important for initial adhesive interactions during cell migration in vivo. Disruption of F-actin increases cell adhesion, whereas disruption of tubulin reduces adhesive interactions in vivo [34]. Therefore, lower cortical tension and higher elastic density of the inner panels may increase the adhesive properties of CaSki, thereby facilitating migration and transformation.

**Fig. 4:**
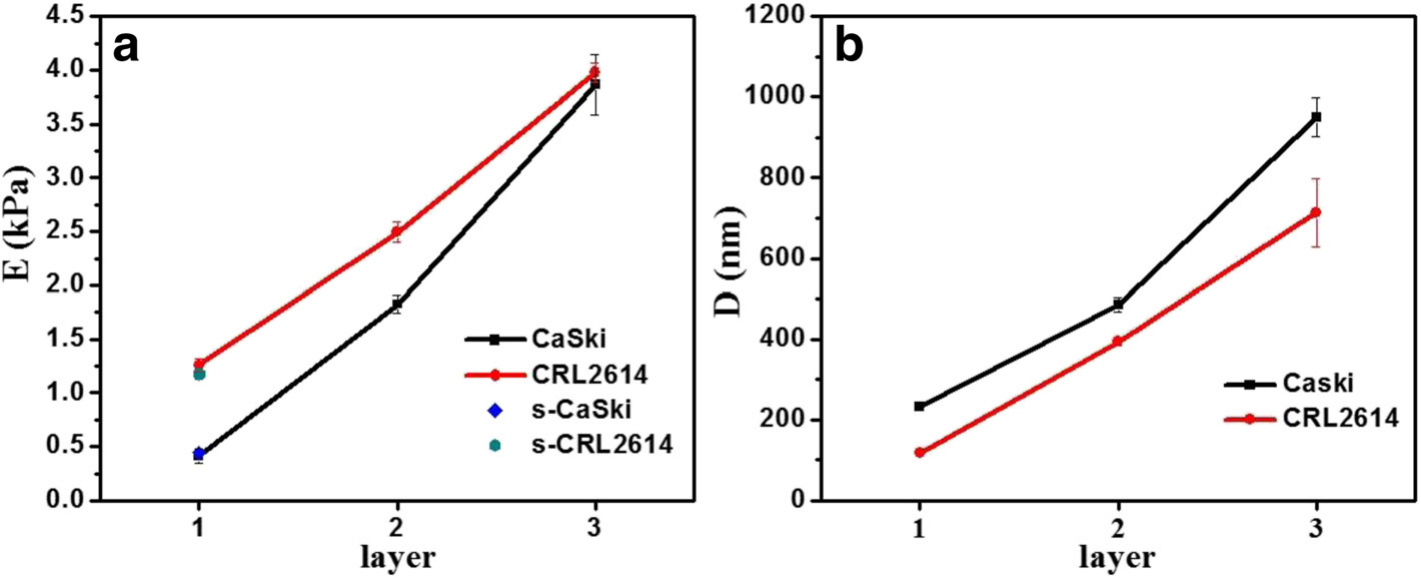
Comparison of elasticity (**a**) and corresponding depth (**b**) of cancer cells and normal cells. *Single plot* represents shallow indentation, and *line* indicates deep indentation. Each *data point* on the graph refers to the most probable values obtained by Gaussian fitting of the corresponding histogram

In order to achieve comparability of results, the most probable values of all analyses were indicated as the mean values (M) ± standard deviations (SD), as shown in Fig. 4. As shown in Fig. 4a, the Young’s elastic modulus of the cells, obtained from AFM of shallow indentation, was consistent with that of the outer panel of deep indentation, thereby validating our improved method. The elasticity curve of CaSki cells was entirely above that of the normal cell line; however, the reverse was observed for the depth curve, indicating that a softer cytoskeleton with shallow distribution may induce, or be induced by, cervical carcinoma. Moreover, the largest *D* value of elastic modulus was observed in the first panels, whereas that of greater depths appeared in the last panel (Fig. 4b). Considering the standard deviation and range of the distribution of data, the greatest difference between the cancerous CaSki cells and normal CRL2614 cells was derived from the difference in elasticity of the first panel. This panel was located at 237~225 nm with a modulus of 0.35~0.47 kPa and at 113~128 nm with a modulus of 1.20~1.32 kPa, for CaSki cells and CRL2614 cells, respectively.

Cervical cancer is the most common malignant tumor among the women in developing countries. The changes in the mechanical properties of the exfoliated cells occur earlier than the changes in cell morphology [34]. The application of AFM to detect cervical exfoliated cells may provide new clues to early detection for cervical cancer and precancerous lesions. Besides, the proposed analysis enable a clear distinction of the three-layered structure, probably giving valuable indications about the position or components of the cytoskeleton in the action of a chemical, mechanical, or physiological process.

## Conclusions

The presented work consolidates previous findings that cells have mechanically multilayered structure. Moreover, heterogeneous structures were found to be highly localized in the three cell panels, in the probing volume of both cell types studied. The inner nuclei zone of both cells exhibited a significantly higher stiffness than the outer cell membrane/cytoplasm, and cancerous cells CaSki possessed a lower whole-cell stiffness and a softer nuclei zone. Similar results were reported by Liu et al. [3030]. Besides, a better differentiated cytoskeleton was found in the inner cytoplasm/nuclei zone of the normal CRL2614 cells, whereas a deeper cytoskeletal distribution was observed in the probing volume of the cancerous counterparts.

Our improved method may be validated by the conventional Hertz–Sneddon method, which is reported to be widely used in the literature. Preliminary results indicate that our method may potentially be applied to improve the detection of cancer cells and provide insights into the pathophysiology of the disease.

## Additional file

Additional file 1:
**Morphology analysis.** The topographies of both cell lines were investigated by AFM imaging to validate and enrich changes induced by cancer qualitatively.
