# Supplementary material for: Discrimination Between Cervical Cancer Cells and Normal Cervical Cells Based on Longitudinal Elasticity Using Atomic Force Microscopy
Source: Nanoscale Res Lett. 2015 Dec 14;10:482. doi: 10.1186/s11671-015-1174-y (PMC4678138; doi:10.1186/s11671-015-1174-y)
Supplement: Additional file 1: — Morphology analysis. The topographies of both cell lines were investigated by AFM imaging to validate and enrich changes induced by cancer qualitatively. [file 11671_2015_1174_MOESM1_ESM.doc]

**Additional file 1**

**Morphology analysis**


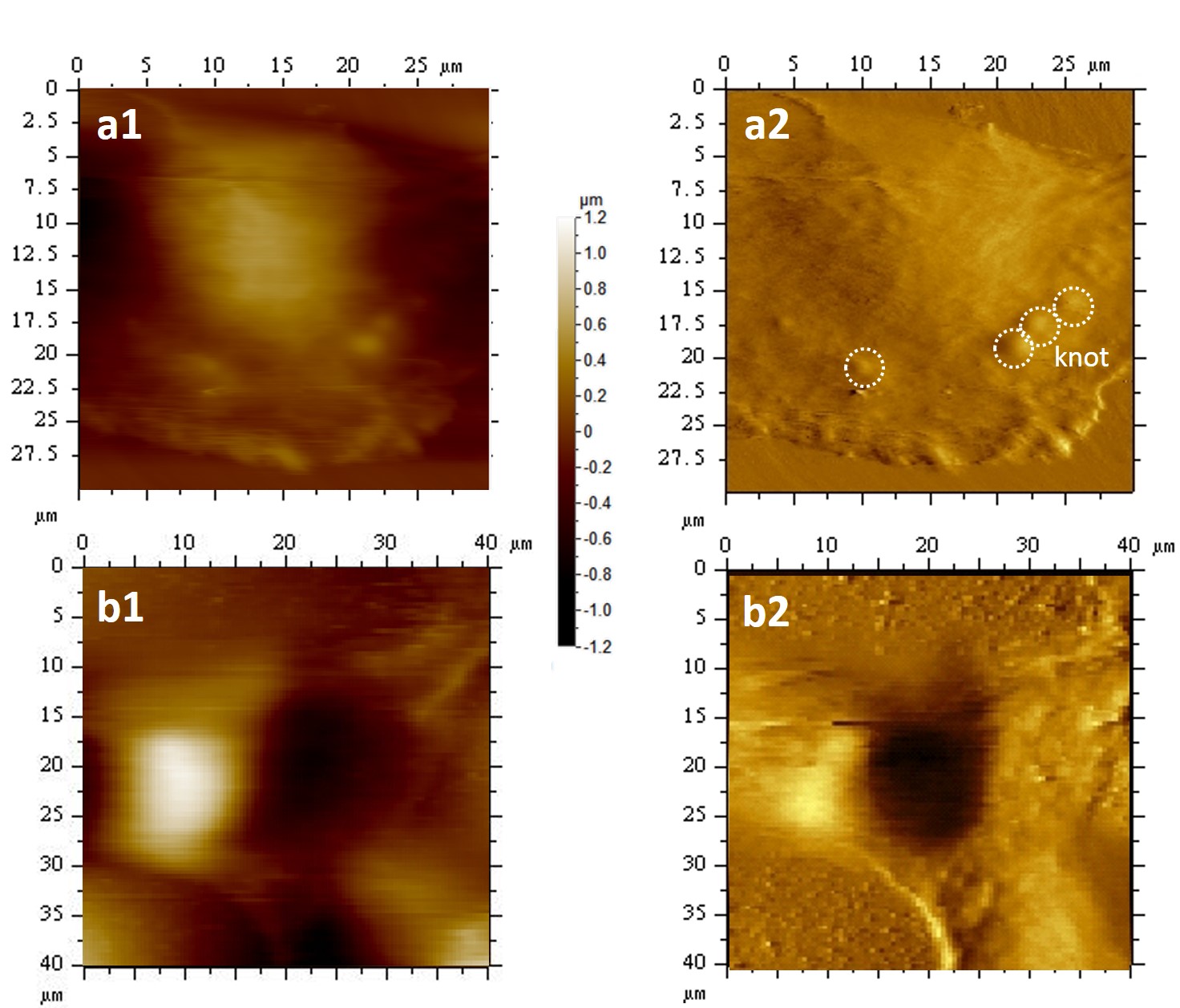


**Figure S1** Typical AFM contact mode imaging. (**a1, b1**) topographic images; (**a2, b2**) corresponding deflection images; (**a1, b1**) for human cervical cancer cells CaSki; (**a2, b2**) for normal cervical epithelial cells CRL2614. Colour coding represents the height: bottom areas are depicted in black, whereas white codes for the top of the sample surface.

The topographies of both cell lines were also investigated by AFM imaging to validate and enrich changes induced by cancer qualitatively. AFM can indent soft membrane during the imaging process, which enables to picture organization of the sub-membrane structures, such as stress fibers. Therefore, distinct organization of cell cytoskeleton and surfacial nanostructures were expected to perform initial evaluation between in normal and cervical carcinoma cells by AFM imaging in medium. The topographic and deflection images of normal cells CRL2614 and cancerous cells CaSki were shown in **Figure** **S1**. The Caski cells comprised disorganized ridges on cell body and well-aligned ﬁlamentous structures at the stretched lamellipodias (**Figure S1a**). Also, it was clearly found in the periphery of CaSki cell some round knots with a diameter of 0.5-2cm scattering, which may be keratin granules, a tendency towards maturation of squamous cell carcinoma^1,2^. Although fuzzy deflection image of live CRL2614 did not provide clear stress structure, we can found that CRL2614 cells had features with no knot, lower nucleus/whole cell ratio and high level of mucus secretion disturbed imaging, relative to that of the CaSki cells.

**References**

1. Mcnairn AJ , Guasch G: Epithelial transition zones: merging microenvironments, niches, and cellular transformation. Eur J Dermatol, 2011. 21: 21-28.
2. Parker J, Vandyke A, Woody E: The prognostic significance of cell type and lesion size in patients with cervical cancer treated by radical surgery. Gynecol Oncol, 1977. 5: 142-151.
